# 3-D chromatin conformation, accessibility, and gene expression profiling of triple-negative breast cancer

**DOI:** 10.1186/s12863-023-01166-x

**Published:** 2023-11-02

**Authors:** Pere Llinàs-Arias, Miquel Ensenyat-Méndez, Javier I. J. Orozco, Sandra Íñiguez-Muñoz, Betsy Valdez, Chuan Wang, Anja Mezger, Eunkyoung Choi, Yan Zhou Tran, Liqun Yao, Franziska Bonath, Remi-André Olsen, Mattias Ormestad, Manel Esteller, Mathieu Lupien, Diego M. Marzese

**Affiliations:** 1 Cancer Epigenetics Laboratory at the Cancer Cell Biology Group, Health Research Institute of the Balearic Islands (IdISBa), 07120 Palma, Spain; 2 Saint John’s Cancer Institute, Providence Saint John’s Health Center, Santa Monica, CA USA; 3 Department of Biosciences and Nutrition, Science for Life Laboratory,, Karolinska Institutet, Stockholm, Sweden; 4 Science for Life Laboratory, Division of Gene Technology, KTH Royal Institute of Technology, Stockholm, Sweden; 5 Department of Biochemistry and Biophysics, Science for Life Laboratory, Stockholm University, Stockholm, Sweden; 6 Josep Carreras Leukaemia Research Institute, Badalona, Barcelona, Catalonia Spain; 7 Centro de Investigación Biomédica en Red Cancer (CIBERONC), 28029 Madrid, Spain; 8 Institució Catalana de Recerca I Estudis Avançats (ICREA), Barcelona, Catalonia Spain; 9 Physiological Sciences Department, School of Medicine and Health Sciences, University of Barcelona (UB), Barcelona, Catalonia Spain; 10 Princess Margaret Cancer Centre, Toronto, Toronto, ON M5G 1L7 Canada; 11 Department of Medical Biophysics, University of Toronto, Toronto, ON M5G 1L7 Canada; 12 Ontario Institute for Cancer Research, Toronto, ON M5G 0A3 Canada

**Keywords:** Epigenetic profiling, Chromatin accessibility, Long-range interactions, RNA levels, ATAC-seq, Hi-C, RNA-seq, MDA-MB-436, MDA-MB-231

## Abstract

**Objectives:**

Triple-negative breast cancer (TNBC) is a highly aggressive breast cancer subtype with limited treatment options. Unlike other breast cancer subtypes, the scarcity of specific therapies and greater frequencies of distant metastases contribute to its aggressiveness. We aimed to find epigenetic changes that aid in the understanding of the dissemination process of these cancers.

**Data description:**

Using CRISPR/Cas9, our experimental approach led us to identify and disrupt an insulator element, IE8, whose activity seemed relevant for cell invasion. The experiments were performed in two well-established TNBC cellular models, the MDA-MB-231 and the MDA-MB-436. To gain insights into the underlying molecular mechanisms of TNBC invasion ability, we generated and characterized high-resolution chromatin interaction (Hi-C) and chromatin accessibility (ATAC-seq) maps in both cell models and complemented these datasets with gene expression profiling (RNA-seq) in MDA-MB-231, the cell line that showed more significant changes in chromatin accessibility. Altogether, our data provide a comprehensive resource for understanding the spatial organization of the genome in TNBC cells, which may contribute to accelerating the discovery of TNBC-specific alterations triggering advances for this devastating disease.

## Objective

Triple-negative breast cancer (TNBC), which accounts for approximately 15–20% of all breast cancer cases, is defined by the absence of estrogen receptor, progesterone receptor, and the lack of human epidermal growth factor receptor 2 (HER2) overexpression and/or amplification [1]. TNBC is associated with a worse prognosis and higher rates of visceral metastases [2]. Matrix metalloproteinases (MMPs) are a family of zinc-dependent endopeptidases involved in the degradation of extracellular matrix components and further invasion, which is the first step of the metastatic cascade [3]. Different MMPs have been associated with poor prognosis in breast carcinomas [4–6]. Given the lower incidence of mutations in breast cancer, other mechanisms, such as epigenetics, may be involved in pathogenesis and progression [7, 8]. For that reason, we aimed to identify epigenetic mechanisms that may dysregulate the expression of MMPs in TNBC.

We found that an insulator element located at chr11:102,730,781–102,736,005 —hereinafter called IE8— is involved in the regulation of gene expression of nine MMP genes. IE8 disruption was performed in TNBC cell lines MDA-MB-231 and MDA-MB-436 through CRISPR/Cas9 transient expression. To gain deeper insights into the molecular mechanisms underlying the consequences of IE8 disruption, we analyzed the chromatin accessibility on our cell line models. We also generated high-resolution maps of three-dimensional chromatin architecture using high‐throughput chromosome conformation capture technology. All analyses were performed in triplicates except duplicates for Hi-C. Additionally, we complemented these datasets with gene expression profiling (RNA-seq) in MDA-MB-231, the cell line that showed more significant changes in chromatin accessibility [9]. These datasets will be a useful resource for researchers focused on TNBC since it is the first study combining Hi-C and ATAC-seq in MDA-MB-231 and MDA-MB-436, two of the most used TNBC cell lines. We believe these datasets represent a valuable resource for a better understanding of TNBC biology.

## Data description

**Fig. 1:**
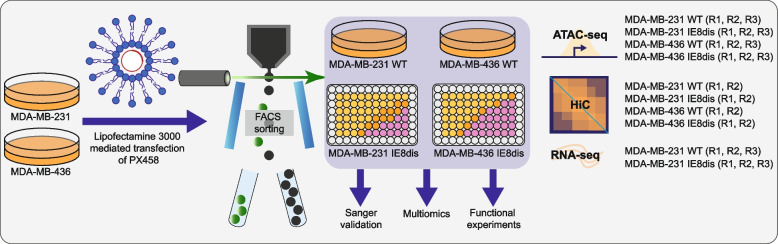
Study design for the generation of insulator element proficient and deficient TNBC cell line models. TNBC cell lines MDA-MB-231 and MDA-MB-436 were considered eligible for the study. They were transiently transfected with PX458 using Lipofectamine 3000. 48 h after transfection, GFP-positive cells were sorted. After model generation functional experiments and multi-omic assays, including ATAC-seq, Hi-C, and RNA-seq were performed

Data files associated with this work are listed in Table 1. The model generation in MDA-MB-231 and MDA-MB-436 TNBC cell lines and the study design are described in Fig. 1 and Data file 2 [10, 11]. TNBC cells were purchased at the American Type Culture Collection (ATCC). Short tandem repeat (STR) analysis was performed at the University of Arizona Genetics Core (Submission UAGC-AM-3154718, Tucson, AZ, USA) to authenticate cell lines before the experiments described in the manuscript. Cells were periodically checked using the MycoAlert Mycoplasma Detection Kit.

**Table 1:** Overview of data files/data sets

Label	Name of data file/dataset	File types (file extension)	Data repository and identifier (DOI or accession number)
Data file 1	Figure 1. Study design	Image file (.jpg)	FigShare (https://doi.org/10.6084/m9.figshare.22820930) [10]
Data file 2	Model generation	Text document (.txt)	FigShare (https://doi.org/10.6084/m9.figshare.22821617) [11]
Data file 3	Figure 2. ATAC-seq quality control (QC)	Image file (.jpg)	FigShare (https://doi.org/10.6084/m9.figshare.22820948) [12]
Data file 4	Figure 3. QC of Hi-C samples	Image file (.jpg)	FigShare (https://doi.org/10.6084/m9.figshare.22821413) [13]
Data file 5	Figure 4. QC of RNA-seq samples and data	Image file (.jpg)	FigShare (https://doi.org/10.6084/m9.figshare.22821437) [14]
Data file 6	Code version	Text document (.txt)	FigShare (https://doi.org/10.6084/m9.figshare.22822115) [15]
Data set 1	ATAC-seq data files	FASTQ files BigWig files	https://identifiers.org/arrayexpress:E-MTAB-12821 [16]
Data set 2	Hi-C data files	FASTQ files mcool files	https://identifiers.org/arrayexpress:E-MTAB-12825 [17]
Data set 3	RNA-seq data files	FASTQ files tablecounts	https://identifiers.org/arrayexpress:E-MTAB-12823 [18]

### Assay for transposase-accessible chromatin using sequencing (ATAC-seq)

ATAC-seq samples were amplified using Nextera barcoded PCR primers as described in Buenrostro et al. [19]. Library generation and sequencing steps were performed following the published protocol by Ryan Corces M, et al. [20]. Amplified libraries were purified and sequenced on a Novaseq6000 (Illumina), 51nt(R1)-10nt(I1)-10nt(I2)-51nt(R2). 33–141 million pairs of 50-bp paired-end read per sample were generated. Reads were adapter-trimmed with Cutadapt and mapped (hg38) using Bowtie 2 [21] with default parameters. Chromatin accessibility peaks were identified with MACS2 with the broad mode [22]. BedTools [23] was used to generate BigWig tracks with a genomic bin size of 50 bp for visualizing chromatin accessibility in the UCSC genome browser [24].

**Fig. 2:**
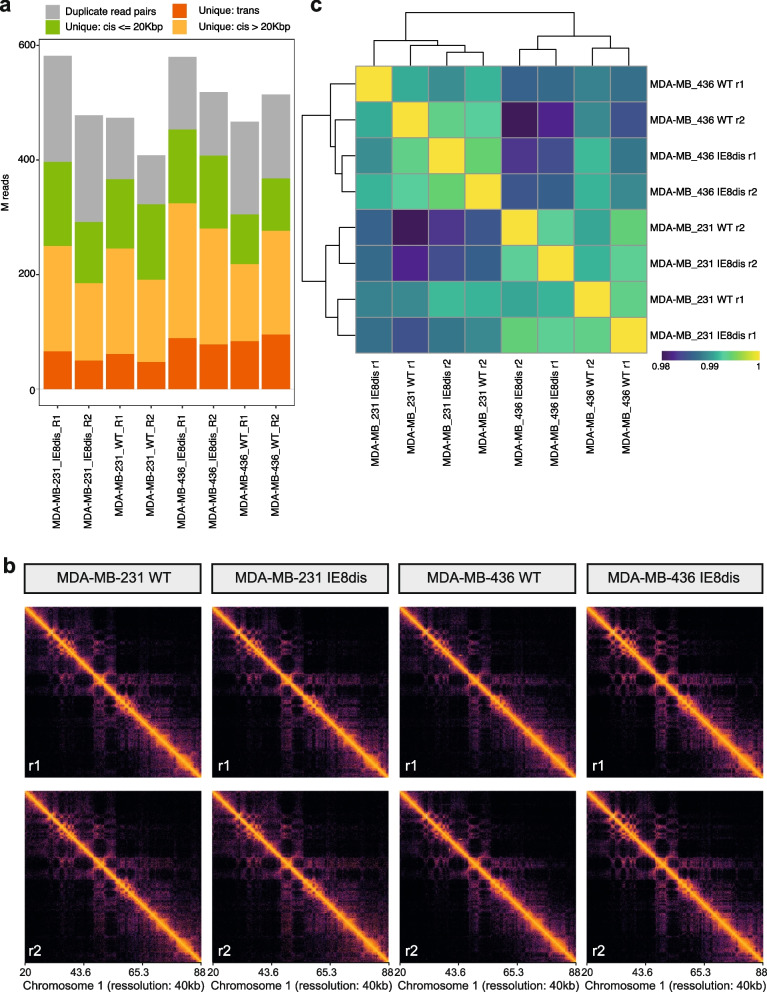
ATAC-seq quality control (QC). **a** Millions of unique and duplicated reads sequenced on each replicate, rounded, and stranded. **b** Fragment length distribution of ATAC-seq reads from a representative sample (MDA-MB-231 WT R1). Most of the reads fall into the nucleosome-free region or mono-nucleosome peak. **c** HOMER peak annotation of genome ontologies from MACS2 called peaks for each replicate. **d** Distance matrix of replicates after DESeq2 processing. **e** Enrichment of ATAC-seq signal around transcription start sites (TSS) in a representative sample (MDA-MB-231 WT R1). Top: aggregated enrichment around all TSSs. Bottom enrichment around individual TSS

Quality control analysis (QC) is summarized in Fig. 2 [18]. Between 14–42 million reads were not duplicated on each replicate. Fragment length distribution was very similar among replicates. The replicate similarity was assessed from clustering by Euclidean distances between DESeq2 rlog values for each sample in the featureCounts file.

### High-throughput chromosome conformation capture (Hi-C)

Hi-C was performed following the manufacturer's protocol from Cantata Bio at the NGI Sweden sequencing facility. Cells were fixed using formaldehyde and disuccinimidyl glutarate (DSG). Afterward, in situ DNase I digestion of the cross-linked chromatin was performed. After digestion, the chromatin fragments were extracted, repaired, and ligated to a biotinylated bridge adapter, and the ends containing the adaptor were ligated close together. Before PCR amplification, biotin-containing fragments were extracted using streptavidin beads. The library prep was done using the NEBNext Ultra II DNA Library Prep (Illumina). Sequencing setup was performed using NovaSeq S4, 151nt(R1)-19nt(I1)-10nt(I2)-151nt(R2). Hi-C reads were analyzed using nf-core/Hi-C pipeline [25] using bowtie2 with local alignment.

**Fig. 3:**
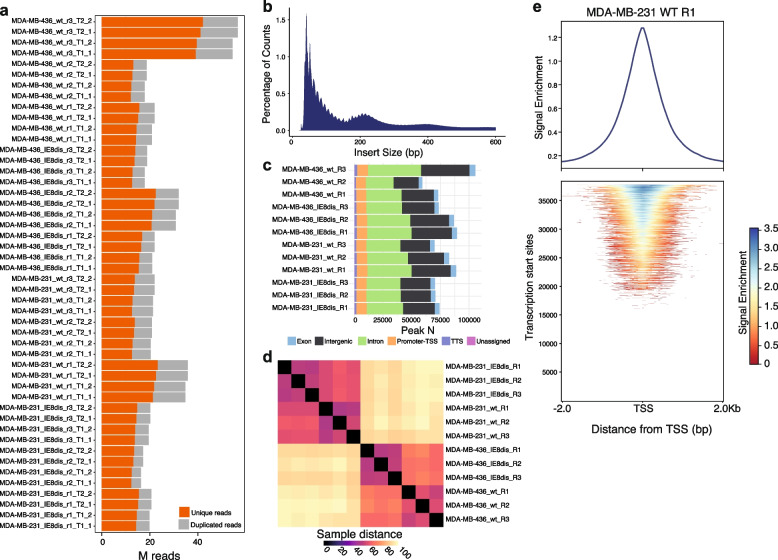
QC of Hi-C samples. **a** Millions of reads sequenced on each replicate. **b** Chromosome interaction heatmaps of all replicates for an exemplary region of chr1 using a 40 kb bin size. **c** Correlation plot of experimental replicates based on the same chr1 region

QC is summarized in Fig. 3 [13]. Different resolution normalized Hi-C-PRO matrices were further generated. 47–95 million reads of unique-trans contacts were identified across replicates. The sample distance matrix was created using chr1 segments with 40kb bin sizes.

### Sample preparation and RNA isolation for expression analysis through RNA-seq

Libraries were created using the Illumina® TruSeq Stranded mRNA Library Prep (Illumina). 500 ng of total RNA were used for mRNA capturing, fragmentation, cDNA synthesis, adapter ligation, and library amplification. Libraries were purified using magnetic beads and sequenced on a NovaSeq 6000 (Illumina) in paired-end mode with a read length of 2 × 100bp. Reads were adapter-trimmed using Fastp software (v0.21.0), mapped (hg38) using HISAT2 (v2.2.0), and sorted using Samtools (v1.10). The read counts table was generated using StringTie (v2.1.4). Table counts were processed using the DEseq2 [26].

**Fig. 4:**
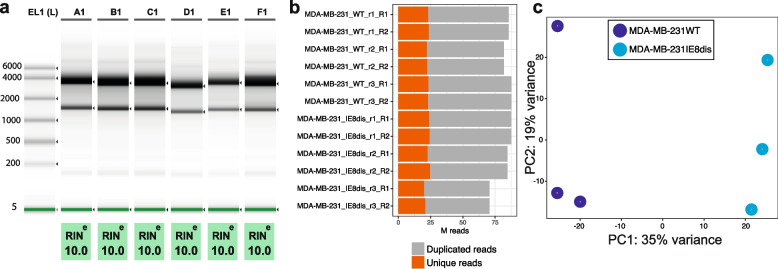
QC of RNA-seq samples and data. **a** RNA integrity number of each replicate, calculated using TapeStation system. **b** Millions of unique and duplicated reads were sequenced on each replicate. **c** Principal component analysis of replicates after DEseq2 processing

QC is summarized in Fig. 4 [14]. The RNA Integrity Number (RIN) for each sample was equal to 10. After sequencing, 70.6–87.7 million pairs of 100-bp paired-end read per sample were generated. Between 20–25 million unique reads were sequenced. Table counts were processed using the DEseq2 [26] to determine the association between samples through a principal component analysis (PCA).

## Limitations

The count of absolute peaks per replicate in ATAC-seq was partially influenced by the more in-depth sequencing that occurred in some replicates, namely MDA-MB-436 WT R3. However, HOMER peak annotation (annotatePeaks.pl) revealed similar peak distribution genome ontology among replicates. RNA-seq was only performed in MDA-MB-231 since we observed a more exacerbated decrease of accessibility after CRISPR/Cas9 disruption of IE8 at this locus. Since we conducted these experiments using only TNBC cell lines, not all the chromatin architecture, chromatin accessibility, and RNA expression features from primary breast samples may have been captured. However, due to the still technical limitations to profile chromatin interactions on tumor tissues, these datasets represent a starting point to discover and explore site-specific chromatin alterations on TNBC.

## Data Availability

The Hi-C raw fastq files and mcool processed files were deposited at the European Genome-phenome Archive (EGA) under the following accession number E-MTAB-12825 [19]. Raw ATAC-seq data, as well as BigWig track files, were deposited at EGA under the accession number E-MTAB-12821 [18]. The RNA-seq transcriptomic data (raw FASTQ and table counts) have been deposited to the EGA repository under the accession number E-MTAB-12823 [20]. A summary of samples and data collection can be found in Data File 1 [10]. The code version can be found here [23] Please see Table 1 for details and links to the data.

## Abbreviations

ATAC-seq: Assay for Transposase-Accessible Chromatin using sequencing
Hi-C: High‐throughput chromosome conformation capture
IE: Insulator element
IE8: Insulator element close to MMP8
MMP: Matrix metalloproteinase
RIN: RNA Integrity Number
RNA-seq: RNA sequencing
TNBC: Triple-negative breast cancer
